# Multiparametric MRI combined with PSA density as a noninvasive rule‐out strategy in active surveillance for prostate cancer

**DOI:** 10.1002/bco2.70079

**Published:** 2025-09-22

**Authors:** Publio Cesar Cavalcante Viana, Marcelo Araújo Queiroz, Fabio Oliveira Ferreira, Adriano Basso Dias, Natally Horvat, Maurício Dener Cordeiro, Claudio Bovolenta Murta, Giuliano Betoni Guglielmetti, Rafael Ferreira Coelho, Leonardo Cardili, José Pontes, William Carlos Nahas, Giovanni Guido Cerri

**Affiliations:** ^1^ Radiology Department University of São Paulo São Paulo Brazil; ^2^ Radiology Department São Paulo Cancer Institute São Paulo Brazil; ^3^ Surgical Oncology Department São Paulo Cancer Institute São Paulo Brazil; ^4^ Joint Department of Medical Imaging, University Health Network, Mt. Sinai Hospital, Women's College Hospital University of Toronto Toronto Canada; ^5^ Department of Radiology Mayo Clinic Rochester Minnesota USA; ^6^ Urology Department São Paulo Cancer Institute São Paulo Brazil

**Keywords:** active surveillance, combined biopsy, ISUP grade, mpMRI, prostate cancer, PSA density, radical prostatectomy

## Abstract

**Objective:**

To evaluate the diagnostic performance of multiparametric MRI (mpMRI), mpMRI combined with PSA density (PSAd) and combined biopsy (CBx) in detecting clinically significant prostate cancer (csPCa) in men undergoing active surveillance, using radical prostatectomy (RP) specimens as the reference standard.

**Patients and Methods:**

In this prospective single‐centre study, 91 patients with low‐risk prostate cancer under active surveillance underwent mpMRI, PSAd measurement, CBx and ultimately RP. mpMRI was reported using PI‐RADS v2.0, and PSAd was dichotomised at 0.12 ng/ml/cm^3^. Diagnostic accuracy was compared using ISUP grade ≥2 and ≥3 thresholds. Radical prostatectomy pathology served as the reference standard.

**Results:**

For detecting ISUP ≥3 cancer, mpMRI combined with PSAd achieved the highest sensitivity (93.3%) and negative predictive value (94.4%). CBx demonstrated the highest specificity (88.2%) and overall diagnostic balance (Youden index = 0.348). mpMRI alone showed intermediate performance. Differences in classification between strategies were statistically significant (McNemar p < 0.001).

**Conclusions:**

mpMRI combined with PSAd provides high sensitivity and negative predictive value for ruling out aggressive prostate cancer, supporting its use as a non‐invasive triage tool in active surveillance. CBx remains the most specific method for histological confirmation. These strategies should be used complementarily to optimise decision‐making in active surveillance protocols.

## 1 INTRODUCTION

Active surveillance (AS) is a widely accepted strategy for managing low‐risk prostate cancer (PCa), aiming to minimise overtreatment while preserving oncologic outcomes.^1^, ^2^, ^3^ Traditional AS protocols incorporate serial PSA testing, digital rectal examination (DRE), and periodic systematic biopsies to monitor disease progression. However, accurately identifying patients who harbour or may develop clinically significant PCa (csPCa) remains a clinical challenge.^2^, ^4^, ^5^, ^6^

Multiparametric magnetic resonance imaging (mpMRI) has emerged as a non‐invasive modality to detect csPCa and guide targeted biopsy (TBx).^7^, ^8^ Despite its increasing adoption, the diagnostic performance of mpMRI compared with systematic biopsy (SBx) and combined biopsy (CBx) remains under debate, particularly in the AS setting.^9^, ^10^, ^11^ PSA density (PSAd) has been proposed as an adjunct marker to improve mpMRI specificity, but its role in refining risk stratification is not yet fully established.^12^, ^13^, ^14^

Integrating mpMRI findings with additional clinical variables, such as PSAd and the number of positive biopsy cores, may enhance prediction of disease progression in AS cohorts.^10^, ^15^, ^16^ Radiogenomic research further supports this approach by demonstrating correlations between molecular profiles and lesion visibility on mpMRI, contributing to a more individualised risk assessment.^6^

Few prospective studies have directly compared mpMRI with biopsy‐based methods using radical prostatectomy (RP) specimens as the reference standard in AS populations.^17^, ^18^ Given the known limitations of biopsy‐based risk classification, including sampling error and tumour heterogeneity,^19^ we hypothesise that mpMRI provides superior diagnostic sensitivity, while PSAd improves specificity.

This study aims to evaluate the diagnostic performance of multiparametric MRI (mpMRI), mpMRI combined with PSA density (PSAd) and combined biopsy (CBx) in detecting clinically significant prostate cancer (csPCa) in men undergoing active surveillance, using radical prostatectomy (RP) specimens as the reference standard.

## 2 MATERIALS AND METHODS

### 2.1 Study design and sample

This prospective, single‐centre study was conducted at a tertiary cancer centre between 2015 and 2020. Ethical approval was obtained from the Institutional Review Board (IRB#: 47949515.4.0000.0065), and all participants provided written informed consent in accordance with the Declaration of Helsinki. The study followed the Standards of Reporting for MRI‐Targeted Biopsy Studies (START guidelines).

A total of 395 patients were consecutively enrolled in the institutional AS protocol. All patients were referred from external institutions without MRI access and were initially diagnosed through random 12‐core biopsy. Only cases confirmed as ISUP grade group 1 upon internal pathology review were included. Confirmatory biopsy consisted of systematic sampling, with MRI‐targeted biopsy selectively performed for PI‐RADS 4–5 lesions.

Eligible patients met criteria for low‐risk PCa (ISUP 1, PSA ≤ 10 ng/ml, clinical stage T1c–T2a, ≤3 positive cores and <50% core involvement on confirmatory biopsy). Exclusion criteria included prior prostate surgery, hormonal therapy, contraindications to mpMRI or TRUS‐guided biopsy or incomplete imaging data. After applying these criteria, 240 patients remained, of whom 91 underwent RP due to disease reclassification or personal choice and were included in the final analysis. All MRIs and biopsies were performed at our institution, with the final exam preceding RP. Patients underwent baseline 3 T mpMRI and confirmatory biopsy within six months of enrolment, followed by semiannual DRE, PSA testing and annual mpMRI. Rebiopsies were performed every 1–2 years or earlier if PSA exceeded 10 ng/ml or if suspicious findings (PI‐RADS 4–5) emerged. Only baseline imaging and biopsy data were analysed. A subset of patients from this cohort has been included in previous publications with different research objectives.^20^, ^21^

### 2.2 Imaging protocol and PSA density

All mpMRI scans were interpreted by a board‐certified uroradiologist with 10 years of experience in prostate imaging. The radiologist was blinded to clinical data and biopsy results. Lesions were scored using PI‐RADS v2.0,^22^ with PI‐RADS 4–5 considered MRI‐positive. Imaging was categorised as low risk (PI‐RADS 1–3) or high risk (PI‐RADS 4–5). PSAd was calculated as PSA (ng/mL) divided by prostate volume (cm^3^) measured on T2‐weighted imaging. Receiver operating characteristic analysis identified 0.12 ng/mL/cm^3^ as the optimal PSAd cutoff. Combined risk classification defined low‐risk cases as PI‐RADS 1–3 with PSAd ≤0.12 ng/mL/cm^3^, and high‐risk cases as PI‐RADS 4–5 or PSAd >0.12 ng/mL/cm^3^.

### 2.3 Standard of reference

CBx included TBx and/or SBx. TBx was conducted for PI‐RADS 4–5 lesions using cognitive fusion with TRUS guidance. SBx consisted of 12‐core sampling from the peripheral and transitional zones. All biopsy samples were reviewed by a single expert uropathologist blinded to clinical and imaging data. RP specimens provided definitive histopathological confirmation and served as the reference standard.

During AS, patients with PI‐RADS 4–5 lesions or CBx upgrading to ISUP ≥2 were reclassified and referred for definitive treatment. Disease progression was defined as PSA > 10 ng/ml, >3 positive biopsy cores, >50% core involvement or ISUP ≥2 on rebiopsy. RP was performed according to protocol criteria or patient decision.

### 2.4 Statistical analysis

Continuous variables were summarised using means, medians, standard deviations and ranges. Categorical variables were presented as frequencies and percentages. To define clinically significant disease, two ISUP grade thresholds were applied: ISUP ≥2 (representing broader risk and early progression) and ISUP ≥3 (representing biologically aggressive cancer).

Diagnostic performance was assessed for mpMRI, mpMRI + PSAd and CBx. Sensitivity, specificity, positive predictive value (PPV), negative predictive value (NPV) and Youden index were calculated for each strategy using both CBx and radical prostatectomy (RP) pathology as reference standards. Emphasis was placed on sensitivity and NPV due to their clinical relevance in safely excluding high‐risk disease and minimising underdiagnosis.

To assess agreement between diagnostic classification and final pathology, reclassification patterns were analysed and categorised as upgrade (low to high risk), downgrade (high to low risk) or no change. Concordance rates were calculated under both ISUP thresholds. The Chi‐square test was used to compare classification patterns between mpMRI and mpMRI + PSAd, particularly when CBx was used as the reference standard.

To compare paired diagnostic methods applied to the same patients, the McNemar test was used. This non‐parametric test evaluates discordant classification outcomes in 2 × 2 contingency tables, providing insight into statistically significant differences in reclassification performance. McNemar tests were performed under both ISUP ≥2 and ≥3 thresholds using RP as the reference, comparing all three strategies (mpMRI, mpMRI + PSAd and CBx). Exact p‐values were reported due to small numbers of discordant pairs in certain comparisons.

Finally, ROC curve analysis was performed to assess the discriminative performance of each method under both thresholds and reference standards. The area under the curve (AUC) and 95% confidence intervals were reported but were interpreted in conjunction with conventional diagnostic metrics, given the overlapping CIs observed.

All statistical analyses were conducted using SPSS version 29 (IBM Corp., Armonk, NY), with a significance level set at p < 0.05.

**FIGURE 1:**
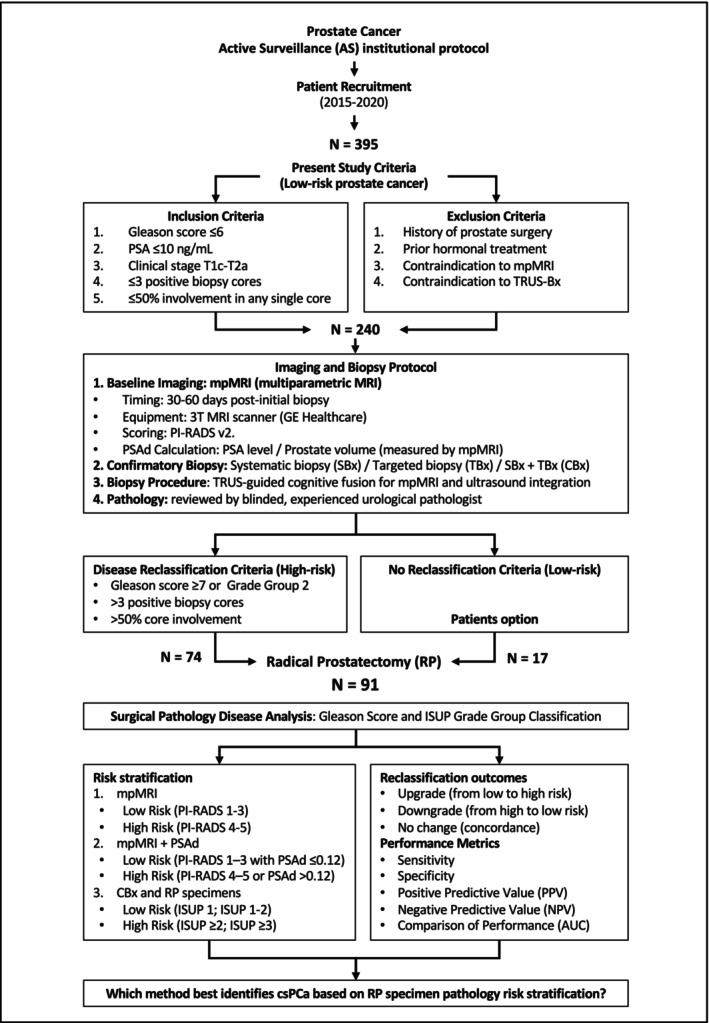
Study design and flowchart of the institutional active surveillance protocol and patient selection for radical prostatectomy (RP). RP = radical prostatectomy; mpMRI = multiparametric magnetic resonance imaging; PSAd = prostate‐specific antigen density; SBx = systematic biopsy; TBx = targeted biopsy; CBx = combined biopsy; ISUP = International Society of Urological Pathology; csPCa = clinically significant prostate cancer.

Figure 1 shows the study design and flow of patients through the institutional active surveillance protocol, including selection, reclassification and surgical pathology comparison.

## 3 RESULTS

Among the 91 patients who underwent RP, the median age was 65 yr (range: 42–77). Regarding self‐reported race, 49 patients (53.8%) identified as White, 28 (30.8%) as Mixed and 14 (15.4%) as Black. The median prostate volume was 50.3 cm^3^ (range: 18.0–177.0; SD 30.88), median PSA was 6.10 ng/ml (range: 2.08–10.0; SD 2.13) and median PSAd was 0.11 ng/ml/cm^3^ (range: 0.03–0.34; SD 0.06).

The median interval between mpMRI and RP was 229 d (range: 4–1036; SD 197.41). This variation was attributed to scheduling delays in the public health system and the COVID‐19 pandemic. Despite this, 83.5% (76/91) of patients underwent surgery within 50–350 d. Based on mpMRI, 28 patients (30.8%) were classified as PI‐RADS 1–3 and 63 (69.2%) as PI‐RADS 4–5. Surgical indication was predominantly based on clinical reclassification (n = 74, 81.3%), while 17 patients (18.7%) elected RP by personal choice.

In addition, the median time from initial prostate cancer diagnosis to radical prostatectomy was 15 months (IQR: 11–24). The interval between confirmatory biopsy and RP was 201 days (IQR: 125–290). All patients underwent mpMRI and confirmatory biopsy within 6 months of AS enrolment, ensuring consistency in baseline assessment.

Table 1 summarises key clinical, radiologic and pathological characteristics, including PI‐RADS classification, PSA density, biopsy findings and ISUP grading from RP specimens.

**TABLE 1:** Patient characteristics and risk stratification based on multiparametric MRI (mpMRI), PSA density (PSAd), combined biopsy (CBx) and radical prostatectomy (RP) surgical specimen pathology.

Variable	Category	N	%
**PI‐RADS Classification**	PI‐RADS 1	1	1.1
	PI‐RADS 2	19	20.9
	PI‐RADS 3	8	8.8
	PI‐RADS 4	43	47.3
	PI‐RADS 5	20	22.0
**PI‐RADS Risk group**	Low Risk (PI‐RADS 1–3)	28	30.8
	High Risk (PI‐RADS 4–5)	63	69.2
**PSAd (ng/mL/cm** ^**3**^ **)**	≤ 0.12	55	60.4
	> 0.12	36	39.6
**mpMRI + PSAd (ng/mL/cm** ^**3**^ **)**	Low Risk (PI‐RADS 1–3 with PSAd ≤ 0.12)	18	19.8
	High Risk (PI‐RADS 4–5 or PSAd > 0.12)	73	80.2
**CBx ISUP Classification**	ISUP 1	47	51.6
	ISUP 2	27	29.7
	ISUP 3	13	14.3
	ISUP 4	3	3.3
	ISUP 5	1	1.1
**CBx Risk group (High Risk; ISUP ≥2)**	Low Risk (ISUP 1)	47	51.7
	High Risk (ISUP ≥2)	44	48.3
**CBx Risk group (High Risk; ISUP ≥3)**	Low Risk (ISUP 1–2)	74	81.3
	High Risk (ISUP ≥3)	17	18.7
**RP Surgical Specimen — ISUP Classification**	ISUP 1	24	26.4
	ISUP 2	52	57.1
	ISUP 3	13	14.3
	ISUP 5	2	2.2
**RP Specimen — Risk group (ISUP ≥2)**	Low Risk (ISUP 1)	24	76.6
	High Risk (ISUP ≥2)	67	23.4
**RP Specimen — Risk group (ISUP ≥3)**	Low Risk (ISUP 1–2)	76	83.5
	High Risk (ISUP ≥3)	15	16.5

Abbreviations: CBx = combined biopsy; ISUP = International Society of Urological Pathology; mpMRI = multiparametric magnetic resonance imaging; PSAd = prostate‐specific antigen density; RP = radical prostatectomy.

### 3.1 Diagnostic performance comparisons

Risk classification agreement was evaluated using ISUP ≥2 and ISUP ≥3 thresholds. When CBx was used as the reference and ISUP ≥2 was applied, mpMRI resulted in 5.5% upgrade, 26.4% downgrade and 68.1% concordance. mpMRI + PSAd yielded 3.3% upgrade, 35.2% downgrade and 61.5% concordance. The difference between the two strategies was not statistically significant (χ^2^ = 1.95; p = 0.378). For ISUP ≥3, both mpMRI and mpMRI + PSAd showed 0.0% upgrade. Downgrades occurred in 51.6% and 62.6% of cases, respectively, with concordance rates of 48.4% and 37.4%. Again, no statistically significant difference was observed (χ^2^ = 2.24; p = 0.326).

When RP was used as the reference and ISUP ≥2 was applied, mpMRI demonstrated 16.5% upgrade, 12.1% downgrade and 71.4% concordance; mpMRI + PSAd showed 11.0% upgrade, 17.6% downgrade and 71.4% concordance; and CBx showed 33.0% upgrade, 7.7% downgrade and 59.3% concordance. These differences were statistically significant (χ^2^ = 16.72; p = 0.002; Table 2).

**TABLE 2:** Reclassification rates (upgrade, downgrade and no change) of multiparametric MRI (mpMRI), mpMRI combined with PSA density (PSAd) and combined biopsy (CBx), using CBx and radical prostatectomy (RP) specimens as reference standards. Results are presented for two risk thresholds: ISUP grade ≥2 and ISUP grade ≥3. Chi‐square (χ^2^) and p‐values refer to comparisons between mpMRI and mpMRI + PSAd.

Ref.	ISUP threshold	Method	Upgrade (%)	Downgrade (%)	No change (%)	Chi‐square (χ^2^)	p‐value
**CBx**	≥2	mpMRI	5.5	26.4	68.1	1.95	0.378
		mpMRI + PSAd	3.3	35.2	61.5		
	≥3	mpMRI	0.0	51.6	48.4	2.24	0.326
		mpMRI + PSAd	0.0	62.6	37.4		
**RP**	≥2	mpMRI	16.5	12.1	71.4	16.72	0.002
		mpMRI + PSAd	11.0	17.6	71.4		
		CBx	33.0	7.7	59.3		
	≥3	mpMRI	3.3	56.0	40.7	65.79	<0.001
		mpMRI + PSAd	1.1	64.8	34.1		
		CBx	8.8	9.9	81.3		

Abbreviations: CBx = combined biopsy; ISUP = International Society of Urological Pathology; mpMRI = multiparametric magnetic resonance imaging; PSAd = prostate‐specific antigen density; RP = radical prostatectomy.

Under the ISUP ≥3 threshold, mpMRI demonstrated 3.3% upgrade, 56.0% downgrade and 40.7% concordance; mpMRI + PSAd yielded 1.1% upgrade, 64.8% downgrade and 34.1% concordance; and CBx showed 8.8% upgrade, 9.9% downgrade and 81.3% concordance. This comparison revealed a statistically significant difference in classification performance (χ^2^ = 65.79; p < 0.001; Table 2).

**FIGURE 2:**
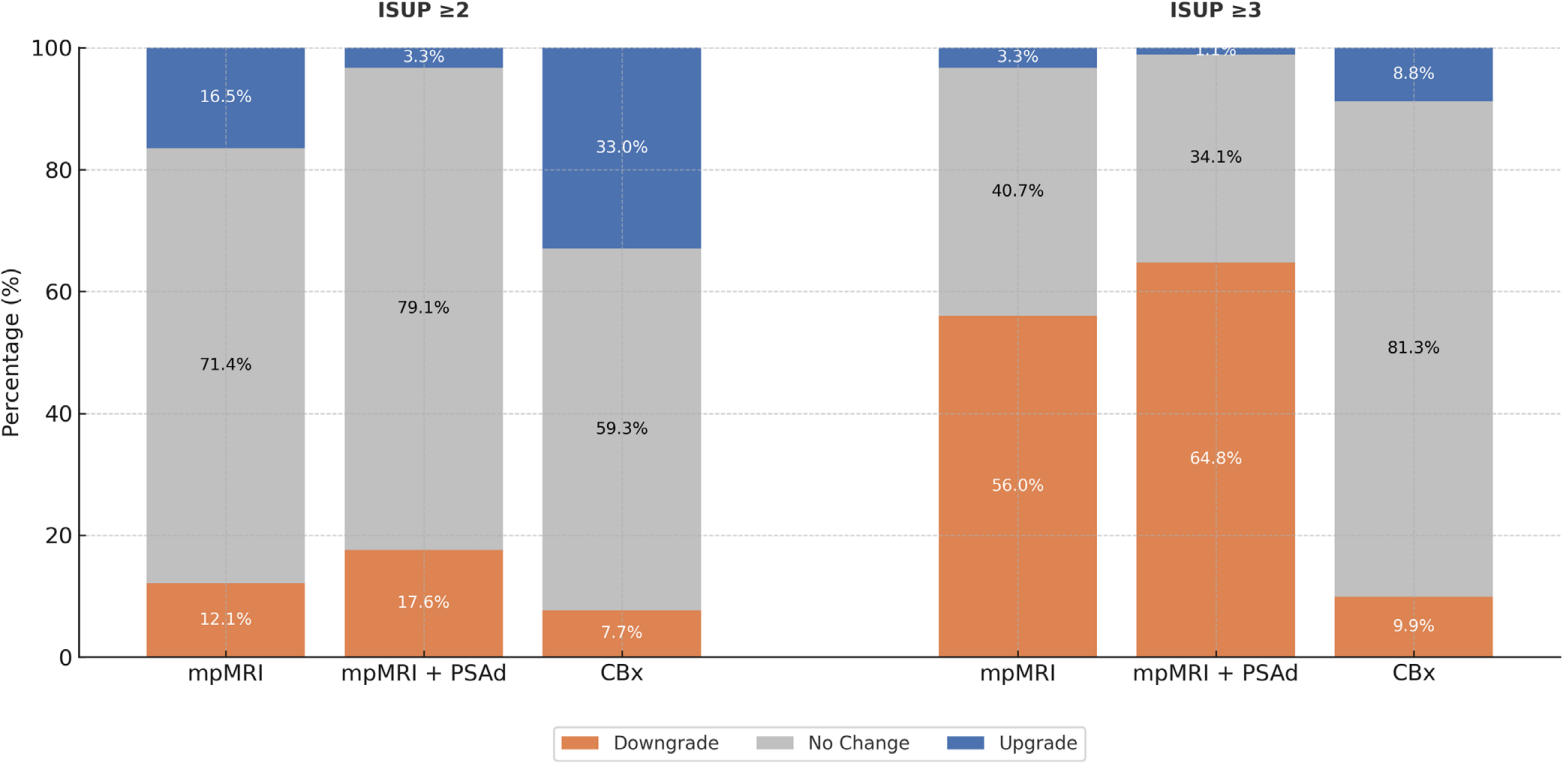
Stacked bar plots of reclassification outcomes (upgrade, downgrade and no change) for mpMRI, mpMRI + PSAd and CBx, using radical prostatectomy (RP) pathology as reference. Results are shown for both ISUP ≥2 and ISUP ≥3 thresholds. mpMRI + PSAd achieved the lowest upgrade rates but was associated with high downgrade rates and limited concordance. In contrast, CBx showed the highest overall concordance and the most balanced classification performance, particularly under the ISUP ≥3 threshold.

Reclassification patterns are visually summarised in Figure 2, which highlights the limited concordance of mpMRI‐based approaches—especially under ISUP ≥3—and the superior classification consistency achieved by CBx.

These findings are further detailed in Table 3, which presents the results of McNemar tests comparing the classification performance of mpMRI, mpMRI + PSAd and CBx under ISUP grade thresholds ≥2 and ≥3, using both CBx and RP as reference standards.

**TABLE 3:** McNemar test results comparing the classification performance of mpMRI, mpMRI + PSAd and CBx under ISUP grade thresholds ≥2 and ≥3, using CBx and RP as reference standards.

Ref.	ISUP threshold	Comparison	Agreement LR ‐ LR	Upgrade LR ‐ HR	Downgrade HR ‐ LR	Agreement HR ‐ HR	p‐value*	Note
CBx	ISUP ≥2	mpMRI vs. mpMRI + PSAd	18	10	0	63	0.002	A
	ISUP ≥3	mpMRI vs. mpMRI + PSAd	23	5	24	39	<0.001	
RP	ISUP ≥2	mpMRI vs. mpMRI + PSAd	15	3	32	41	<0.001	A
		mpMRI vs. CBx	18	10	0	63	0.002	B
		mpMRI + PSAd vs. CBx	23	5	24	39	<0.001	A
	ISUP ≥3	mpMRI vs. mpMRI + PSAd	15	3	32	41	<0.001	A
		mpMRI vs. CBx	18	10	0	63	0.002	B
		mpMRI + PSAd vs. CBx	23	5	24	39	<0.001	A

* McNemer test.

A ‐ Adding PSAd to mpMRI increases high‐risk classification, potentially enhancing sensitivity.

B ‐ mpMRI increases the number of patients classified as high risk.

### 3.2 Overall diagnostic accuracy

ROC analysis was conducted to assess the discriminative performance of mpMRI and mpMRI combined with PSAd, using CBx and RP specimens as reference standards, under ISUP grade thresholds ≥2 and ≥3. For ISUP ≥2, when CBx was used as the reference, the AUC for mpMRI was 0.688 (95% CI: 0.578–0.798), while mpMRI + PSAd yielded an AUC of 0.626 (95% CI: 0.511–0.740). Using RP as the standard, the AUCs were 0.659 (95% CI: 0.526–0.792) for mpMRI, 0.592 (95% CI: 0.453–0.731) for mpMRI + PSAd and 0.630 (95% CI: 0.502–0.758) for CBx. For ISUP ≥3, the AUCs using CBx as the reference were 0.687 (95% CI: 0.569–0.804) for mpMRI and 0.620 (95% CI: 0.488–0.752) for mpMRI + PSAd. With RP as the reference, mpMRI achieved an AUC of 0.564 (95% CI: 0.412–0.717), mpMRI + PSAd 0.579 (95% CI: 0.432–0.725) and CBx 0.675 (95% CI: 0.508–0.840).

When RP was used as the reference standard, all three diagnostic strategies were evaluated. For ISUP ≥2, mpMRI and mpMRI + PSAd demonstrated AUCs of 0.659 (95% CI: 0.526–0.792) and 0.592 (95% CI: 0.453–0.731), respectively, while CBx achieved an AUC of 0.630 (95% CI: 0.502–0.758). For ISUP ≥3, the AUCs were 0.564 (95% CI: 0.412–0.717) for mpMRI, 0.579 (95% CI: 0.432–0.725) for mpMRI + PSAd and 0.675 (95% CI: 0.508–0.840) for CBx. Although CBx yielded higher AUCs in both thresholds, the overlapping confidence intervals indicate that these differences may not be statistically significant.

These results are summarised in Table 4, which presents AUC values with 95% confidence intervals and qualitative interpretations for all strategies.

**TABLE 4:** AUC values (95% CI) and qualitative interpretation for mpMRI, mpMRI + PSAd and CBx in detecting clinically significant prostate cancer, using CBx or RP as reference standards under ISUP thresholds ≥2 and ≥3.

Ref.	ISUP threshold	Method	AUC (95% CI)	Interpretation
CBx	ISUP ≥2	mpMRI	0.688 (0.578–0.798)	Highest AUC in group
		mpMRI + PSAd	0.626 (0.511–0.740)	Overlapping CI – not significantly different
	ISUP ≥3	mpMRI	0.687 (0.569–0.804)	Highest AUC in group
		mpMRI + PSAd	0.620 (0.488–0.752)	Overlapping CI – not significantly different
RP	ISUP ≥2	mpMRI	0.659 (0.526–0.792)	Highest AUC in group
		mpMRI + PSAd	0.592 (0.453–0.731)	Overlapping CI – not significantly different
		CBx	0.630 (0.502–0.758)	Overlapping CI – not significantly different
	ISUP ≥3	mpMRI	0.564 (0.412–0.717)	Overlapping CI – not significantly different
		mpMRI + PSAd	0.579 (0.432–0.725)	Overlapping CI – not significantly different
		CBx	0.675 (0.508–0.840)	Highest AUC in group

CI = confidence interval; RP = radical prostatectomy; AUC = area under the curve; ISUP = International Society of Urological Pathology; PSA = prostate‐specific antigen.

### 3.3 Diagnostic metrics with CBx and RP as references

When CBx was used as the reference standard, mpMRI alone demonstrated good sensitivity but modest specificity. Under the ISUP ≥2 threshold, it yielded a sensitivity of 88.6%, specificity of 48.9%, PPV of 61.9% and NPV of 82.1%, resulting in a Youden index of 0.376. Adding PSAd increased sensitivity to 93.2% but reduced specificity to 31.9%. In this case, PPV and NPV were 56.2% and 83.3%, respectively, and the Youden index declined to 0.251—indicating reduced overall diagnostic balance despite the gain in sensitivity.

For the ISUP ≥3 threshold, both mpMRI and mpMRI + PSAd achieved 100% sensitivity and NPV. However, specificity decreased to 37.3% for mpMRI and 24.0% for mpMRI + PSAd. The corresponding PPVs were 25.4% and 21.9%, and the Youden indices were 0.373 and 0.240, respectively.

When RP was used as the reference standard, a different performance profile emerged. Under the ISUP ≥2 threshold, mpMRI alone showed 77.6% sensitivity and 54.2% specificity, with a PPV of 82.5% and NPV of 46.4%, yielding a Youden index of 0.318. The addition of PSAd improved sensitivity to 85.1%, but at the cost of reduced specificity (33.3%), and yielded a PPV of 78.1%, NPV of 44.4% and a lower Youden index of 0.184.

In this same setting (ISUP ≥2 with RP as reference), CBx showed the lowest sensitivity (55.2%) but the highest specificity (70.8%), along with a PPV of 84.1%, NPV of 36.2% and a Youden index of 0.261, suggesting better specificity at the expense of sensitivity.

Under the ISUP ≥3 threshold and using RP as the reference, mpMRI achieved a sensitivity of 80.0% and specificity of 32.9%, with a low PPV (19.0%) but relatively high NPV (89.3%), resulting in a Youden index of 0.129. mpMRI + PSAd further increased sensitivity to 93.3%, but specificity dropped to 22.4%. The PPV remained low (19.2%), while NPV rose to 94.4%, with a Youden index of 0.157.

In contrast, CBx exhibited the most balanced performance in this context, achieving 46.7% sensitivity and 88.2% specificity. Its PPV was 43.8% and NPV 89.3%, with a Youden index of 0.348—indicating superior diagnostic balance compared to mpMRI‐based strategies.

A comparative analysis of these findings underscores the distinct diagnostic strengths of each strategy. While mpMRI + PSAd consistently achieved the highest sensitivity and NPV—key features for confidently excluding high‐grade disease—CBx demonstrated superior specificity and overall diagnostic balance, particularly under the ISUP ≥3 threshold. These complementary attributes reflect the clinical trade‐offs between maximising detection and minimising overtreatment.

**FIGURE 3:**
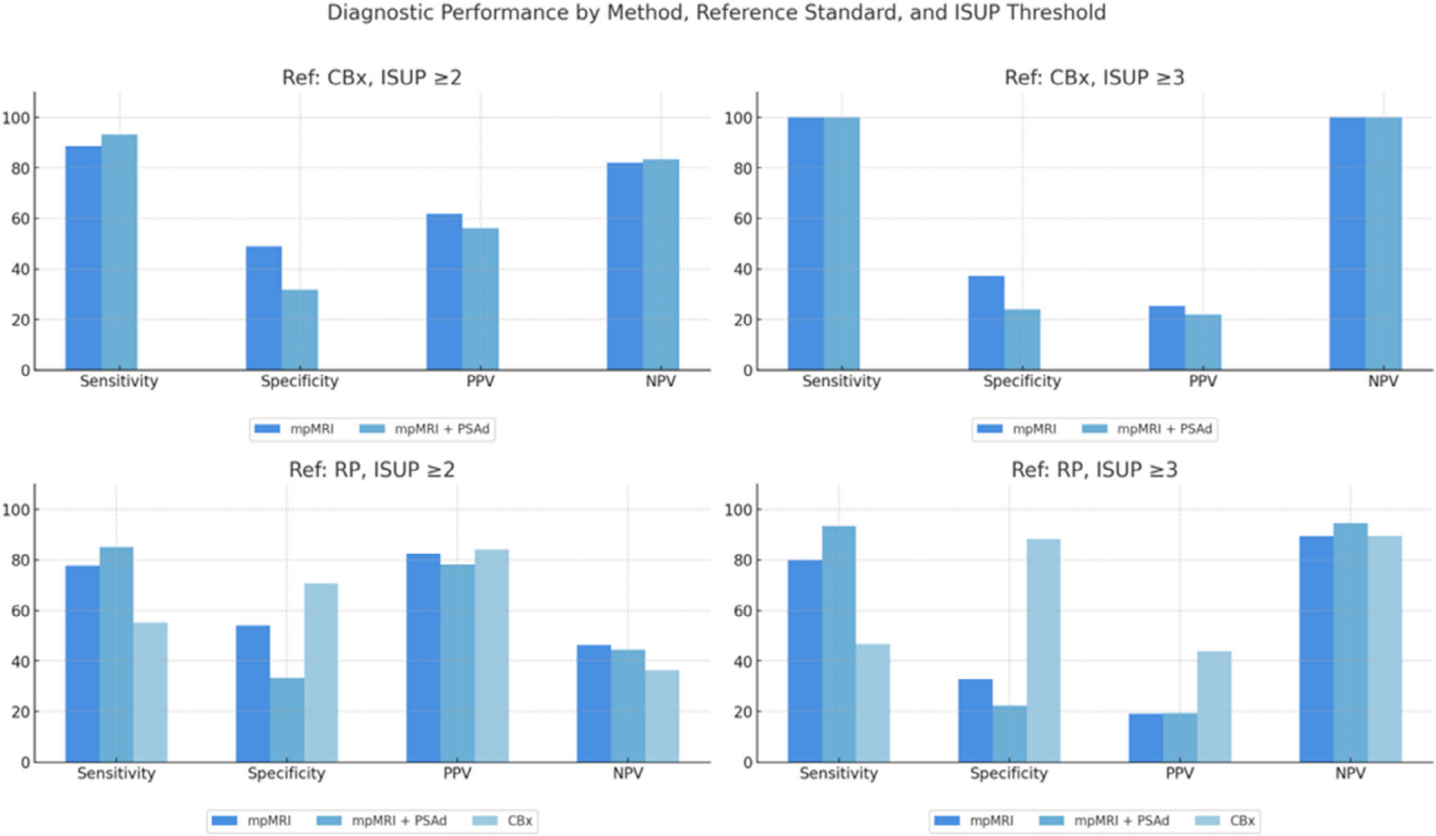
Diagnostic performance of mpMRI, mpMRI + PSAd and CBx across reference standards (CBx and RP) and ISUP thresholds (≥2 and ≥3). Bar charts display sensitivity, specificity, positive predictive value (PPV), negative predictive value (NPV) and Youden index for each method under four different conditions: CBx as the reference with ISUP ≥2 and ≥3 (top row), and RP as the reference with ISUP ≥2 and ≥3 (bottom row). Shades of blue represent the different diagnostic strategies.

Table 5 presents a detailed comparison of performance metrics for each method across thresholds and reference standards, and Figure 3 provides a visual overview of these results.

**TABLE 5:** Diagnostic performance of mpMRI, mpMRI combined with PSA density (PSAd) and combined biopsy (CBx) for detecting clinically significant prostate cancer. Radical prostatectomy (RP) and combined biopsy (CBx) were used as reference standards. Performance is reported for two thresholds of clinical significance: ISUP ≥2 and ISUP ≥3.

Ref.	ISUP threshold	Method	Sensitivity (%)	Specificity (%)	PPV (%)	NPV (%)	Youden index
CBx	≥2	mpMRI	88.6	48.9	61.9	82.1	0.376
		mpMRI + PSAd	93.2	31.9	56.2	83.3	0.251
	≥3	mpMRI	100.0	37.3	25.4	100.0	0.373
		mpMRI + PSAd	100.0	24.0	21.9	100.0	0.240
RP	≥2	mpMRI	77.6	54.2	82.5	46.4	0.318
		mpMRI + PSAd	85.1	33.3	78.1	44.4	0.184
		CBx	55.2	70.8	84.1	36.2	0.261
	≥3	mpMRI	80.0	32.9	19.0	89.3	0.129
		mpMRI + PSAd	93.3	22.4	19.2	94.4	0.157
		CBx	46.7	88.2	43.8	89.3	0.348

Abbreviations: CBx = combined biopsy; ISUP = International Society of Urological Pathology; mpMRI = multiparametric magnetic resonance imaging; NPV = negative predictive value; PPV = positive predictive value; RP = radical prostatectomy.

## 4 DISCUSSION

In this prospective study evaluating AS patients who underwent RP, we compared the diagnostic performance of mpMRI, mpMRI + PSAd and CBx using pathological outcomes as the reference standard. Overall, mpMRI‐based strategies, particularly when combined with PSAd, provided the highest sensitivity (93.3%) and negative predictive value (NPV up to 94.4%) under the ISUP ≥3 threshold. These characteristics are essential in active surveillance settings, where the primary concern is the risk of underdiagnosing clinically significant prostate cancer. The ability of mpMRI + PSAd to confidently exclude high‐grade disease supports its use as a frontline triage tool to reduce unnecessary biopsies and overtreatment.^5^, ^8^, ^9^, ^13^

In contrast, CBx exhibited the highest specificity (88.2%) and the most balanced diagnostic performance. This suggests greater accuracy in confirming significant disease once suspicion has been raised. However, its lower sensitivity (53.3%) and high upgrade rate compared to imaging‐based strategies raise concerns regarding tumour under‐sampling. These findings underscore the limitations of CBx as a standalone method in AS, especially when the clinical goal is to confidently rule out aggressive cancer while minimising procedure‐related morbidity.^10^, ^13^

The diagnostic performance of CBx in the full 240‐patient AS cohort was previously assessed in a prospective study from our group,^20^ which demonstrated that patients with negative mpMRI (PI‐RADS ≤3) and PSA density <0.15 ng/mL^2^ had a very low risk of upgrading on confirmatory biopsy (NPV 97.9%). These findings support the selective omission of CBx in this subgroup. In contrast, upgrading was strongly associated with PI‐RADS 4–5 lesions (adjusted OR 16.2), reinforcing the role of mpMRI in risk stratification. The present study, which focused on the 91 patients who proceeded to radical prostatectomy, complements that earlier analysis by using whole‐mount pathology as reference. Our findings are also consistent with the recent study by Bhanji et al.,^23^ which showed low upgrading rates in MRI‐negative patients, though their results still supported routine CBx due to upgrading observed in systematic cores. Together, these data highlight the value of integrating PSA density and mpMRI findings to refine biopsy strategies in AS.

When RP was used as the definitive reference, imaging strategies showed higher concordance than CBx under the ISUP ≥2 threshold (71.4% vs. 59.3%), although CBx performed best in ISUP ≥3 concordance (81.3%). These patterns reinforce the trade‐offs between sensitivity and specificity depending on the threshold used. Imaging‐based methods demonstrated a consistent ability to identify patients unlikely to harbour ISUP ≥3 cancer, with very low upgrade rates (1.1–3.3%) and high NPVs, reaffirming their role in safely delaying or avoiding definitive treatment in selected patients.^2^, ^4^, ^5^, ^6^

Our findings align with the PROMIS trial, which defined clinically significant disease as Gleason ≥4 + 3 or maximum cancer core length ≥6 mm, demonstrating that mpMRI had superior sensitivity (93%) and NPV (89%) compared to TRUS‐guided biopsy.^5^ In our cohort, similar results were observed when mpMRI + PSAd was applied under a comparable ISUP ≥3 threshold, reinforcing the clinical validity of these criteria in defining biologically aggressive disease.

The observed improvements in diagnostic sensitivity with the addition of PSAd support previous studies that highlighted the incremental value of volume‐adjusted PSA in enhancing mpMRI performance.^11^, ^12^, ^24^, ^25^, ^26^ Notably, the integration of PSAd resulted in improved sensitivity without increasing the upgrade rate, suggesting a net clinical benefit in the context of risk stratification.

While mpMRI + PSAd emerged as the optimal model for ruling out csPCa, the choice of diagnostic strategy should be tailored to clinical intent. In scenarios prioritising exclusion of high‐grade cancer, mpMRI + PSAd may serve as a gatekeeper to reduce biopsy burden. Conversely, in patients with high clinical suspicion, CBx remains the most robust tool for histologic confirmation.

This study has limitations. All imaging was interpreted by a single, highly experienced uroradiologist, potentially limiting external validity. The single centre design and lack of inter‐reader variability assessment further constrain generalisability. Additionally, our cohort consisted solely of patients undergoing RP, which may not reflect the broader AS population and could introduce selection bias. Nonetheless, the use of RP pathology as the gold standard strengthens the reliability of our comparisons. Additionally, the PSAd threshold (0.12 ng/ml/cm^3^) was not externally validated. Finally, we acknowledge that the time from initial mpMRI to RP varied substantially (median 229 days, range 4–1036). This heterogeneity reflects real‐world constraints, including the COVID‐19 pandemic and limited surgical capacity in the public health system, which often delayed access to radical treatment. Although all patients underwent routine follow‐up with semiannual PSA and DRE and annual mpMRI between 2015 and 2020, re‐biopsies were performed every 1–2 years or earlier based on clinical indications. These factors may have introduced variability in follow‐up duration but also reflect the practical realities of managing AS cohorts in resource‐constrained settings. Similarly, to our study, future research should adopt a prospective design, use prostatectomy specimens as the reference standard, and explore longitudinal imaging‐based monitoring while integrating molecular or AI‐derived tools to optimise risk stratification.^27^, ^28^

## 5 CONCLUSIONS

In conclusion, mpMRI combined with PSAd achieved the highest sensitivity and NPV, favouring its use as a non‐invasive strategy to safely exclude aggressive disease in men under AS. CBx, while more invasive, remains the most accurate modality for confirming ISUP ≥3 cancer. The integration of imaging and histologic methods rather than choosing one over the other should guide decision‐making in AS protocols.

## AUTHOR CONTRIBUTIONS

*Study concept and design*: Viana, Queiroz, Ferreira. *Data acquisition*: Viana, Ferreira, Cordeiro. *Analysis and interpretation*: Viana, Ferreira, Horvat, Murta. *Drafting of the manuscript*: Viana, Queiroz, Ferreira, Dias, Horvat. *Critical revision of the manuscript*: All authors. Supervision: All authors.

## CONFLICT OF INTEREST STATEMENT

The authors declare no conflicts of interest.
